# The presence of Y chromosome leads to the difference of cancer in patients with different sexes

**DOI:** 10.1002/mco2.468

**Published:** 2024-01-12

**Authors:** Yingqi Kuang, Wenhuan Sun, Long Zhang

**Affiliations:** ^1^ International Biomed‐X Research Center Second Affiliated Hospital of Zhejiang University School of Medicine Zhejiang University Hangzhou China; ^2^ Institutes of Biology and Medical Science Soochow University Suzhou China; ^3^ Cancer Center Zhejiang University Hangzhou China

**Keywords:** cancer, sex, Y chromosome

## Abstract

(A) In the case of LOY, the proportion of T cells and suppressor macrophages rises and T cells enhance the expression of TOX, eventually leading to the metastasis of the tumor. (B) KRAS^mut^ enhances the gene expression of *KDM5D*, leading to a decreased expression of the AMOT and TAP1/2 downstream of *KDM5D*. Thereby, the cell−cell junction and antigen presentation are affected. All elements in Figure 1 are original

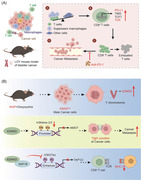

.

1

In two recent articles published simultaneously in “Nature,” Abdel‐Hafiz et al.^1^ and Li et al.^2^ found that the presence of the Y chromosome leads to differences in cancer in patients with different types of bladder cancer. Li et al. found that Y‐chromosome loss (LOY), a common phenomenon in bladder cancer, is associated with poor prognosis. LOY can promote T‐cell depletion, leading to an inhibitory microenvironment and a more aggressive tumor (Figure 1A). In contrast, Abdel‐Hafiz constructed a mouse colorectal cancer (CRC) model, and his research team found that cancer metastasis was higher and the prognosis was worse in male CRC mice carrying the oncogenic KRAS mutation (KRAS*) than that in females (Figure 1B). To some extent, both findings illustrate that the presence of Y chromosome leads to differences in cancer in patients with different sexes.

**FIGURE 1:**
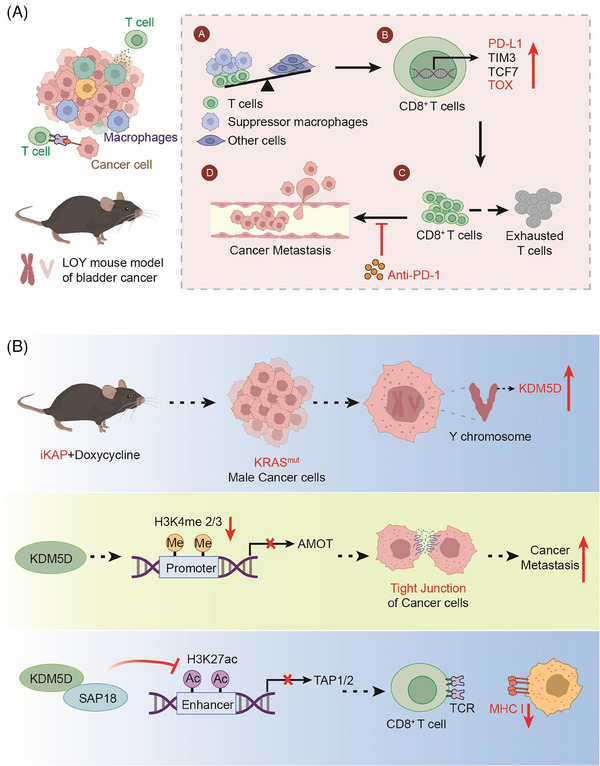
(A) In the case of LOY, the proportion of T cells and suppressor macrophages rises and T cells enhance the expression of TOX, eventually leading to the metastasis of the tumor. (B) KRAS^mut^ enhances the gene expression of *KDM5D*, leading to a decreased expression of the AMOT and TAP1/2 downstream of *KDM5D*. Thereby, the cell−cell junction and antigen presentation are affected. All elements in Figure 1 are original.

Scientists have long recognized that sex influences the incidence, clinical outcome, and biology of cancer. For example, male patients tend to have a worse prognosis than female patients for most cancer types.^3^ Whereas, the specific mechanisms underlying these changes are not well understood. Notably, in these two studies, researchers working on the Y chromosome shed some light on the reason. On the one hand, Li et al. identified a relationship between Y chromosome deletion (LOY) and poor prognosis after the surgical resection of bladder cancer in clinical patients. They found that Y_high_ had a higher survival rate than Y_low_. At the same time, *KDM5D* expression was associated with it. Then, the authors confirmed that low expression of *KDM5D* and *UTY* was associated with poor prognosis using TCGA RNA data, TCGA DNA sequencing data, and mosaic alteration detection for LOY (MADLOY).

To further investigate how LOY causes cancer metastasis, the authors first cultured the MB49 bladder‐cancer cell line (with natural LOY) in vitro and found that the loss of the Y chromosome did not affect the growth of cancer cells. Interestingly, when the authors subcutaneously injected the cancer cells into mice, the Y^−^ cancer cells grew twice as fast as the Y^+^ cancer cells. This suggests that the lack of Y chromosome gene expression was due to LOY. Subsequently, the authors found that LOY in immunocompromised Rag2^−/−^ ‐IL2RG^−/−^ and male C57BL/6 mice affected cancer cell growth only when the immune system was normal. Similar results were obtained by repeating the above experiments using the CRISPR system to knock out the Y chromosome. Therefore, the authors conducted further studies focusing on the immune system.

To identify the discrepancy associated with immunity, the researchers performed transcriptome sequencing of mouse tumors and found that Y^−^tumors had an increased proportion of total CD8^+^T cells and immunosuppressive macrophages, along with increased PD‐L1 expression. Analysis of urothelial bladder cancer sequencing results from the TCGA database also supported this conclusion. In the Y_low_ group, the expression of the immune checkpoint molecules PD‐L1, LAG3, and TIM3 in CD8^+^T cells and the T‐cell exhaustion marker (TOX1^4^) were increased. It suggested that LOY promotes T‐cell depletion in the tumor microenvironment, leading to tumor metastasis.

On the other hand, Abdel‐Hafiz found that the presence of upregulated genes on the Y chromosome was associated with adverse outcomes in CRC.

First, investigators used a mouse CRC model in which an inducible transgene encoding the oncogenic mutant KRAS^G12D^ and the conditional null alleles of Apc and Trp53 tumor suppressors (designated iKAP) showed that the oncogenic mutant KRAS (KRAS*) CRC had higher metastatic rates and worse outcomes in males than in females. Furthermore, based on the corresponding Colorectal Cancer Subtype Consortium analysis of a comprehensive CRC dataset, the authors showed that male KRAS*CRC patients had lower survival rates than female KRAS*CRC patients. Therefore, a more in‐depth study was conducted to understand the reason behind this phenomenon.

The Y‐chromosome genomic protein demethylase *KDM5D* was found to be transcriptionally upregulated driven by KRAS*‐mediated STAT4 transcription factor activation. Notably, the upregulation of STAT4 leads to enhanced binding to the promoter region of *KDM5D*, which ultimately leads to the upregulation of histone *KDM5D* expression. This further validates the role of the KRAS*‐STAT4‐KDM5D pathway in CRC metastasis. On this account, the following question arises: Where does *KDM5D* regulate its substrate to cause tumor metastasis? Finaly, *H3K4me2/3*, a gene downstream of *KDM5D*, was detected using chromatin immunoprecipitation with sequencing (chip‐seq) analyses.

At the same time, RNA‐seq expression profiles showed that *KDM5D* inhibited *H3K4me2/3* activation, and *AMOT* ^5^ and *PKP1* were found to be the key genes in cell−cell junction maintenance. Loss of cell−cell junctions can lead to cancer‐cell spread, which is a critical step in early metastasis. The upregulation of *KDM5D* can inhibit the effect of *H3K4me2/3*, which leads to the inhibition of *AMOT* expression through the inhibition of its downstream gene, the *AMOT* promoter. Thereby, the cell−cell junctions are affected, which leads to tumor metastasis.

On the other hand, cross‐species molecular and transcriptomic analyses also revealed the repression of major histocompatibility complex class I regulators. Chip‐seq data showed that *KDM5D* deletion increased H3K27ac, an active enhancer mark. Therefore, the researchers conducted a further study and found that *KDM5D* can inhibit the action of this super‐enhancer, thereby downregulating the expression of its downstream substrate, TAP1/2. TAP1/2 inhibits CD8^+^T cell‐induced antigen presentation and leads to cancer cell metastasis. These conclusions were also demonstrated using ovalbumin‐treated iKAP cells for antigen presentation. Consequently, KRAS*‐STAT4‐mediated upregulation of the Y chromosome *KDM5D* contributes significantly to sex differences in KRAS*CRC by disrupting cancer‐cell adhesion properties and tumor immunity. Additionally, it provides an actionable therapeutic strategy for men with KRAS*CRC to reduce the risk of metastasis. This also indicates to a certain extent that the presence of the Y chromosome leads to adverse consequences in tumors.

Although they studied the relationship between chromosomes and cancer from different directions, they all focused on the Y chromosome. On the one hand, Abdel‐Hafiz found that LOY can lead to changes in the tumor microenvironment and finally lead to poor prognosis by using genomic and transcriptomic studies. Alternatively, they also observed that Y^−^ tumors have a high sensitivity to anti‐PD‐1 treatment, which partially provides the context for our subsequent drug research. Nonetheless, there is still a lot of research that we need to go into. First, LOY occurs in a variety of cancer types, its clinical and biological significance is not clear. On the other hand, through the combination of bioinformatics and cell biology, Li found that KRAS*‐STAT4‐mediated upregulation of Y chromosome KDM5D contributes substantially to the sex differences in KRAS* CRC by means of its disruption of cancer cell adhesion properties and tumor immunity, providing an actionable therapeutic strategy for metastasis risk reduction for men afflicted with KRAS* CRC. Whereas, sex has a profound impact on cancer incidence, spectrum, and outcome, the molecular and genetic basis of this sex difference is not well defined. This also provides some direction for our subsequent research.

All in all, these are two great works, but there are still some unknowable things we need to study further. At the same time, we can also study more disease differences from chromosome differences in follow‐up studies, and provide new methods for more disease research.

## AUTHOR CONTRIBUTIONS

Yingqi Kuang and Wenhuan Sun contributed equally to this work. Yingqi Kuang and Wenhuan Sun generated the images and wrote the manuscript. Long Zhang provided some modifications. All the authors have read and approved the final manuscript.

## CONFLICT OF INTEREST STATEMENT

The authors declare no conflict of interest.

## ETHICS STATEMENT

No ethical approval was necessary for this work.

## Data Availability

No data were used for the research described in this highlight.
